# E-commerce adoption among micro agri-business enterprise in Longsheng, China: The moderating role of entrepreneurial orientation

**DOI:** 10.3389/fpsyg.2022.972543

**Published:** 2022-11-09

**Authors:** Hanfang Li, Guateng Liow, Shengjun Yuan

**Affiliations:** ^1^School of Digital Economy, Guilin University of Electronic Technology, Guilin, China; ^2^School of Business, Shandong Xiehe University, Jinan, China

**Keywords:** e-commerce adoption, TOE framework, entrepreneurial orientation, poverty alleviation, rural China

## Abstract

E-commerce in agri-business enterprises is a potent force in eradicating rural poverty in China. However, it has not reached its potential as many of the agri-business enterprises in rural areas are slow in re-thinking their distribution channel and implementing e-commerce. This study utilizes the multidimensionality of the Tornatsky and Fleisher Technology-Organization-Environment (TOE) framework to provide insights into why rural agri-business enterprises are not adopting e-commerce strategies. Specifically, it examines the moderating role of entrepreneurial orientation (EO) on the relationship between the sub-variables in the three contexts and e-commerce adoption. Empirical data from 192 micro agri-business enterprises were analyzed using PLS-SEM. The results show that relative advantage, organization readiness, competitive pressure, and government support have a direct positive impact on e-commerce adoption except for cost. The results also show that EO does not have a significant role in moderating the variables in the organizational context and environmental context. EO only moderated the relationship between relative advantage in the technology context and e-commerce adoption. This study advances research on e-commerce adoption by highlighting the importance of the owners' EO as a moderator between TOE factors and e-commerce adoption. It suggests that entrepreneurship must be pursued vigorously among agribusiness owners in rural China to enhance the adoption of e-commerce.

## Introduction

Due to the massive rural population in China, the Chinese government embarked on an aggressive market-oriented reform to develop rural e-commerce as a vital tool to support poverty alleviation in China's rural areas. In Feb 2021, on the 100 th anniversary of the founding of the Communist Party of China (CPC), Chinese President Xi Jinping declared that China has achieved the “miracle” of eradicating extreme poverty measured against a standard equivalent to US$2.30 per person per day applied to rural areas. Nearly 100 million people have been lifted out of poverty. Chinese rural enterprises have been widely recognized to represent the most dynamic force in the Chinese economy and exemplify the fundamental nature of China's socialist market economy (Pooj et al., 2009). Despite the huge success stories reported in the many e-commerce villages or more popularly known as the Taobao villages, the potential of e-commerce has not been exploited fully, particularly among agri-business enterprises in very poor rural counties in China (Li and Wang, 2018).

The transformation from a traditional business to a digital business is a daunting task and fraught with many challenges despite the very high penetration rate of online shopping in China (Akram et al., 2021). As of December 2021, approximately 81.6% of Internet users in China had shopped online, up from 79.1% by the end of 2020 (Ma, 2021). Although, the literature is replete with studies pertaining to the barriers of e-commerce, such as Raphael et al. (2016), Rohit and Tripti (2019), and Mousa (2021), most of these studies relate to large, medium-sized enterprises and studies on the factors influencing micro enterprises are few and far between. Besides, most of these studies are descriptive and lack a powerful theoretical groundwork (Haryanti and Subriadi, 2020). Thus, there are gaps among the influencing factors of e-commerce adoption in micro agri-business enterprises in poor rural China. This paper seeks to address the challenges influencing the e-commerce adoption among farmers running micro businesses in West China, where most of the poorest counties are located as many of the targets of the IT adoption study are concentrated in developed provinces at the Eastern region (Su et al., 2021) or other countries (Mousa, 2021; Nasution et al., 2021). Hence, this paper attempts to understand the idiosyncratic nature of micro businesses in rural China to provide a more nuanced understanding of the institutional context, the individual disposition of the owners of these micro enterprises, and their perception of the challenges and barriers impeding the adoption of e-commerce in a poverty-stricken county, Longsheng, located in Southern China, in the northeast of Guangxi, China.

Notably, the challenges face by poor counties differ in several contextual differences (both organizational and environmental), and the multidimensionality of the Technology-Organization-Environment (TOE) framework is considered as having better explanatory power than a model that covers only one dimension (Molla and Licker, 2004). In addition, TOE has a flexible structure to vary the characteristics of the different contexts (technological, organizational, environmental, and individual) and many salient factors remain unexplored. Therefore, the TOE framework is found to be an appropriate framework for this study. Apart from the factors in the TOE context, Premkumar (2003) suggested that individual factors should also be included in the study of the adoption of e-commerce. Most of the rural agri-business enterprises in Longsheng, China are very small and they have to rely on the capability of their owner-manager as a source of sustainable competitive advantage to enhance the enterprise's performance. Hence, this study suggests that the owner's personal characteristic is pertinent to rural entrepreneurship and seeks to examine if the owners of this rural agribusiness possess an entrepreneurial orientation or if they are merely farmers running a small agribusiness. In addition, the current study suggests that the capability embedded in the owners will enable the enterprises to generate a disposition toward entrepreneurial activity in their enterprises and such entrepreneurial disposition can moderate the relationship between the three elements of the enterprise's context and adoption. Therefore, the objectives of this study are to examine the perceptions of the owners on their perception of the different factors within the TOE context of the adoption of e-commerce among rural agri-business enterprises and how their perception of their entrepreneurial orientation moderates the relationships among the TOE factors and the adoption of e-commerce.

In pursuing the objectives, the paper is organized as follows: First, it briefly reviews the literature on the nature of micro agri-business enterprises in rural China and e-commerce entrepreneurship in rural China. Second, it discusses the theoretical framework and past related research in this area to examine the key variables imperative to the success of rural e-commerce that may increase the adoption rate of e-commerce in rural China. Third, it describes the research methodology followed by a detailed analysis of the results from the survey. Finally, it discusses the findings and conclusions and future research direction.

## Review on micro agri-business enterprises and e-commerce in rural China

In this part, the literature on micro agri-business enterprises and e-commerce entrepreneurship in rural China is reviewed. The micro enterprise and micro agri-business enterprises are defined and their characteristics are analyzed. E-commerce, especially the rural e-commerce development in China is expounded. In addition, the barriers to e-commerce adoption in rural China are analyzed in the context of rural entrepreneurship.

### The nature of micro agri-business enterprises in rural China

In China, many micro enterprises have benefitted from the broad reach of the internet and there are many success stories (Akram et al., 2020). However, this is not often the case for micro businesses in poorer counties, the struggle of rural micro enterprises in utilizing e-commerce to reach wider markets remains an uphill task for these micro businesses. Many have yet to capitalize on e-commerce.

This paper looks at the adoption of e-commerce as a sustainable marketplace by owners of micro enterprises in one of the poorest counties in China by examining the factors or hurdles that influence the adoption of e-commerce by owners of micro agri-business enterprises. According to the National Bureau of Statistics in 2019, 85.3% of businesses in China were micro enterprises and only 13.2 and 1.3% were small enterprises and medium-sized enterprises based on the definition shown in Table 1. Exact limits vary between industries respectively.

**Table 1:** Micro enterprise in China for the different industry types.

**Industry type**	**Micro enterprise**
Manufacturing	Revenue < 3 million RMB
	Employee < 20
Wholesale	Revenue < 10 million RMB
	Employees < 5
Retail	Revenue < 1 million RMB
	Employee < 10

Source: Ministry of Industry and Information Technology (MITT), National Bureau of Statistics, National Development and Reform Commission, Ministry of Finance, China (2011).

Micro agri-business enterprises refer to businesses in agriculture, forestry, animal husbandry, and fishery. In Longsheng, the micro agri-business enterprises are mainly micro businesses engaged in simple processing with a family as a unit. The business mainly uses family residential as places for production, and family members with some work personnel typically make up the workforce. In the past, China's agricultural production has been conducted in a fragmented small-manner and the production of agricultural products has remained highly dispersed. To maximize the potential of e-commerce for a sustainable business, these micro enterprises must accept, adopt, and use the e-commerce technology fully and move up the maturity level of adoption in terms of the functionalities of e-commerce. Most micro enterprises have not recognised that e-commerce can achieve the benefits of a wider market reach, although some have adopted the basic characteristics of e-commerce, such as WeChat mini programs, WeChat stores, where farmers can become an entrepreneur to build a network of reliable, repeat customers (Akram et al., 2017, 2018a,b; Guo and Gao, 2017).

### Rural entrepreneurship and e-commerce

It is clear that promoting entrepreneurial activities is an important strategy to drive stimulating the dissemination of economic activities and promoting sustainable development not just in urban areas but more crucially in rural areas. It has a strong effect on economic growth and job creation and plays an important role in sustainable development and a decisive role in increasing employment and social integration of the population in rural areas (Boghean and State, 2020). However, not enough studies looked at rural entrepreneurship due to a lack of access to data and empirical analyses. It is not surprising that most of the theoretical and empirical contributions of the geography of entrepreneurship are predominantly confined to cities and metropolitan regions (Pato and Teixeira, 2016). Lekhanya (2018) defined rural entrepreneurship as enterprises operating in a rural environment disconnected from primary metropolitan sites that function under extremely complex and turbulent business conditions presented by remote and underdeveloped areas, where local production is primarily committed to subsistence farming. Such disconnects from the primary metropolitan sites that show higher entrepreneurial dynamism probably explained the difference in the rate of entrepreneurship that occurs between rural and urban areas. However, there are also studies (such as Delfmann et al., 2014; Eriksson and Hane-Weijman, 2017) that show how entrepreneurship can be successful in rural regions. Closer to home, in China, instead of providing subsidies, e-commerce is leveraged to create a sustainable business model in the rural area and it has been shown to be able to promote inclusive growth by reducing information costs and help build a more open and transparent market environment. Today, rural e-commerce has greatly enhanced the stability of the supply chain of the produce from the farms and helped to increase the farmers' income rapidly (Ma et al., 2020). However, despite the significant positive effect of e-commerce in increasing the income of farmers, there is evidence that uneven regional development has impacted the adoption of e-commerce. A recently published study suggests that some of the promises by e-commerce giants, such as Rural Taobao have been oversold (Couture et al., 2021) despite having more than 3,000 rural marketplaces branded as “Taobao Villages” based on their high concentration of online sales (AliResearch, 2019). A huge difference exists among different villages; particularly among the impoverished region in the western area and special poor areas such as Tibet, Ningxia, Gansu, Xinjiang, Guizhou, Guangxi, and Yunnan. Mainstreaming of e-commerce, particularly in the western poor rural areas in China not only presents immense growth potential and untapped income streams for e-commerce; it can also encourage many migrants to move back to these poor villages that they left behind and be micro-entrepreneurs in rural areas.

### Study area

Longsheng is a county in the northeast of Guangxi, China, and it is under the administration of Guilin. Longsheng has the highest population of ethnic minorities in northern Guilin and was the first autonomous county established in the south-central region. It is 87.2% mountainous with a forest coverage rate of 79.12%. It covers a total area of 2,538 square km, and the villages and towns are highly dispersed. The total population was 186 thousand and the agricultural population made up 80.8%. In 2021, the per capita net income of rural households was 15,408 RMB, below the average provincial level of 26,727 RMB.

Agriculture is the economic foundation of Longsheng, and agri-business enterprises play an important role to promote the development of agriculture. The micro agri-business enterprises in Longsheng are characterized by very simple production, processing, management, storage, and other processes that are all done in the same place; the degree of standardization is also very low with low scientific and technological content; low profit, and weak competitiveness. The rapid development of e-commerce has broadened the sales channels of agricultural products. Despite the government's great attention to the development of e-commerce, the e-commerce adoption ratio remains low. Only 10% of the micro agri-business enterprises in Longsheng adopted e-commerce.

## Theoretical background, research framework, and hypotheses

In this part, the theoretical background of the research is given first, and then the past related studies are reviewed, the barriers and challenges of e-commerce adoption are analyzed, the research framework is designed, the key variables are selected, and the hypotheses are proposed finally.

### Theoretical background

Research on rural e-commerce and its relationship with organizational learning of micro enterprises is becoming more urgent and important. Existing literature on the adoption by micro enterprises has not been adequately addressed; the lack of theoretical frameworks and empirical evidence to understand how micro agri-business enterprises can realize e-commerce benefits amidst their multi-pronged contextual challenges. This study draws its inspiration from Zhu and Kraemer's (2005) integration of two models: TOE and the Resource Based View (RBV) theory toward understanding the relationship among technology, environment, organizational context, and the individual entrepreneurial orientation of the individual owners in creating and sustaining the benefits of e-commerce. Given the distinct nature of the offerings of these micro agri-business enterprises, the difference in the use and belief about the values of these resources is plausible. Thus, it is expected that there will be differences between types of organizations and industries in the adoption of e-commerce. These organizations differ in the readiness of their institutional infrastructure, capability, and individual owners' entrepreneurial orientation to organize and integrate the different resources into an enterprise to establish the enterprise's legitimacy and distinctive contribution (Busenitz et al., 2001).

RBV helps explain how enterprises can create value by combining heterogeneous resources that are economically valuable, difficult to imitate, or imperfectly mobile across enterprises. Such creation of value is required to effectively use and realize the benefits of e-commerce in expanding the market reach of these micro enterprises. Furthermore, drawing from the resource-based view, the entrepreneurial orientation of the micro enterprise owners serves as an important resource to combine or enhance the resources and capabilities of the enterprise and utilize them in an effective way toward realizing the benefits of e-commerce.

Technology-Organization-Environment, the multi dimension framework proposed by Tornatzky and Fleischer (1990) has been widely applied to investigate factors influencing e-commerce adoption (Rohit and Tripti, 2019; Abdulkarem and Hou, 2021). TOE framework allows the researcher to vary the characteristics of the three distinct aspects of an enterprise's context that influence the process by which it adopts and implement e-commerce: (1) Technological context, (2) Organizational context, and (3) Environmental context. This framework is one of the most dominant, valid, and specific for enterprise context adoption (Awa et al., 2017) due to its proposed generic factors that influence adopters' and non-adopters technology adoption. To further enhance the explanatory and predictive capability of the TOE framework, the entrepreneurial orientation (EO) of the enterprise owners or the decision makers as an additional driver/context as the decision maker's assumption about the future and the consequences of adopting e-commerce can influence the adoption rate; particularly in very small enterprises that are run by owner and the enterprise's strategic and tactical focus is largely shaped by the peculiarity of minds of the powerful coalition (Awa et al., 2015; Nasution et al., 2021). It is impossible to separate the owners of the micro agri-business enterprises from their enterprises since the decisions of the enterprises are all made by them, no matter their daily activities or future investments. This paper suggests that the capability embedded in the owner will enable the enterprise to generate a disposition toward entrepreneurial activity in their enterprise. Given that individual characteristics were rarely espoused in the TOE framework despite the propositions by scholars such as Thong (1999), Hamidreza and Hamideh (2012), and Awa et al. (2015) that enterprise's strategies are directly shaped by the perceptual idiosyncrasies of the decision maker as well as their feelings of the technology's usefulness and effortlessness, which ultimately drive motivation and attitude toward e-commerce adoption (Awa et al., 2017).

### Past related research

As discussed above, the paper was motivated by the limited studies related to e-commerce adoption by micro agri-business enterprises, especially in impoverished rural areas. In comparison with larger companies, the adoption of e-commerce by micro, small and medium enterprises (SMEs) lags far behind. Many researchers have identified the factors inhibiting the adoption of e-commerce by SMEs in developing countries such as India, Malaysia, and Indonesia but less in China. In addition, there were very few studies on the moderating effect of the EO of owners on an enterprise's decision to adopt or not adopt e-commerce in rural areas.

Ahmad et al. (2015) studied the factors affecting e-commerce adoption among SMEs in Malaysia and found that the main factors include perceived relative advantage, perceived compatibility, managers'/owners' knowledge and expertise, management characteristics, and external change agents. Rita and John (2015) investigated the influencing factors of e-commerce adoption by SMEs in Indonesia with the TOE framework and found that perceived benefits, technology readiness, owners' innovativeness, owners' IT ability, and owners' IT experience are the determinant factors. Seng et al. (2018) investigated the influence of competitive pressure on the adoption of e-commerce among SMEs in West Malaysia and verified the positive effect of competitive pressure on the adoption of e-commerce. Rohit and Tripti (2019) conducted a study on Handicraft SMEs in India and found that awareness, human resources, strategy and market forces e-readiness have some influence on the adoption of e-commerce. From the research in different counties, it can be seen that the barriers and challenges are different for the SEMs in different countries, and the determinant factors of e-commerce adoption are related to the characteristics of the SMEs in different countries.

### Research model and conceptual framework

This paper proposed that the adoption of e-commerce at the organizational level can be influenced by its technological, and organizational context within the TOE framework and the individual owners' entrepreneurial orientation.

#### Technological context

Technological context factors include the attributes of the technologies, both internal and external technologies that may have an effect on the decision to adopt. According to Rogers (1995), there are five attributes: relative advantage, compatibility, complexity, trialability, and observability. Other authors such as Doherty and Fulford (2006) found that concern for network security issues can inhibit adoption. The initial cost associated with the adoption of e-commerce, cost of purchasing the e-commerce software were also highlighted as major issues influencing an enterprise, particularly very small enterprises' initiatives to adopt e-commerce (Jahanshahi et al., 2013). Given the size of the micro enterprises, relative advantage and cost (affordability) are perceived to influence the uptake of e-commerce.

#### Organization context

Organizational context refers to the descriptive characteristics of the organization, such as the enterprise size and scope, complexity of the managerial structure, and quality of its human resources (Pearson and Grandon, 2005; Rohit and Tripti, 2019). In Longsheng, the micro agri-business enterprises are relatively simple in terms of organizational structure as they are mostly small, and the type of business typically covers only the production and marketing of agricultural and sideline products. Most of these micro agri-business enterprises have a very simple flat structure and there is no distinct management role; therefore, the nature, size, type of enterprises, and managerial structure are not considered as most of the agri-business enterprises in China are locally owned family farms and the size and managerial structure are the same. Given the stage of development of these micro agri-business enterprises, this study suggests that organization readiness and technology readiness are important variables.

#### Environmental context

Environmental context relates to the operational facilitators and inhibitors, including competitive pressure, government support, technology support, and trading partners' readiness (Sattam and Sami, 2011). Many of these adoption predictors are assumed to apply more to the large organization than to SMEs (Awa et al., 2010; Abdulkarem and Hou, 2021) and may not be relevant to micro enterprises. The liability of smallness represents a key challenge for these small enterprises. In such micro enterprises, tactical and strategic activities are characterized by *ad hoc*, idiosyncratic solutions, and less formality. The support provided by the government in the form of incentive policies and measures from the government can help facilitate adoption. Competitive pressure was selected as an important influence factor of e-commerce in many works of literature (Wang and Ahmed, 2009; Hamidreza, 2012; Ismail, 2013; Rita and John, 2015; Ahmed, 2018; Rohit and Tripti, 2019). It is found to increase the likelihood of IT adoption (Seng et al., 2018).

## Entrepreneurial orientation

Entrepreneurial orientation refers to the way of entrepreneur or business owner looks and searches for any new possibility or innovation during environmental uncertainties that can be implemented in the organization (McMullen and Shepherd, 2006) which is in line with Covin and Wales (2012) view that EO is having the courage of decision making. For micro businesses, the EO of owners is regarded as a main resource and capability for small enterprises' growth *via* the adoption of facilitating technology such as e-commerce. It is therefore pertinent to integrate the EO of the business owners as one of the critical success factors facilitating the adoption of e-commerce. EO is used widely to describe the behavioral patterns of the owners of the enterprises, and its moderating role in the relationship between the influencing factors and business performance is well reported (Abebe, 2014; Najafi and Abdulsalam, 2016; Abdullah and Hassan, 2018; Ade et al., 2020). The importance of EO on enterprise performance is widely recognized, and the moderating role of other relationships between entrepreneurial skill and entrepreneurial intention has been verified (Najafi and Abdulsalam, 2016). Therefore, this study introduces EO as a moderator in the relationships to better understand the barriers impeding e-commerce adoption.

**Figure 1:**
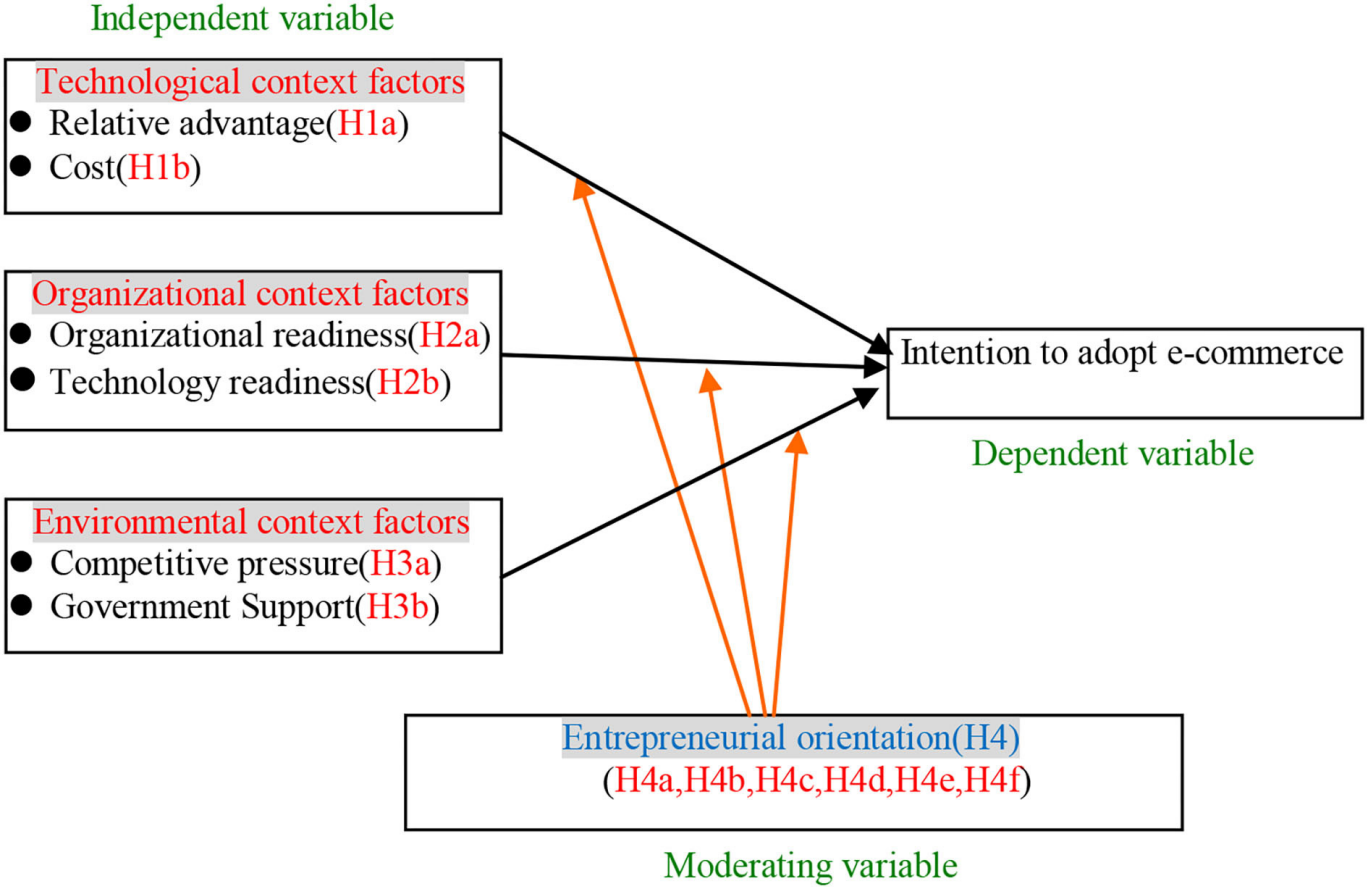
The proposed conceptual framework.

To summarize, relative advantage of the technology, initial cost of implementing e-commerce, organization readiness, technology readiness, governmental support, competitive pressures, and the EO of owners are perceived to influence the adoption of e-commerce. These critical factors were factored in the proposed conceptual framework as per Figure 1.

### Hypotheses development

#### Technology context

Relative advantage and cost are used to reflect the technology context. Rogers (1995) defined relative advantage as the degree to which an innovation is perceived as being better than the idea it overtakes which is consistent with Chalermsak and Nitaya (2003) finding that as the level of understanding of the relative advantage of e-commerce is higher, the higher chances for the enterprise to adopt e-commerce. Various studies (Tan and Teo, 2000; Jati et al., 2017; Ali et al., 2018) have reported a positive relationship between relative advantage and e-commerce adoption. Hence the following hypothesis is proposed.

***H1a**: Relative advantage has a positive effect on the intention to adopt e-commerce in micro agri-business enterprises in Longsheng*.

Cost is the money and time invested in the innovation process. For e-commerce adoption, the cost may include the investment in the network, computer, data storage, servers, and other software/hardware devices and the training time on the staff. Many studies have found that cost is the biggest problem faced by SMEs in e-commerce adoption (Ismail and Osman, 2012; Adi and Sudarsono, 2016). This is not surprising in China as the average incomes of smallholder farmers are just about one-third of their urban counterparts (Grain, 2012). Hence, it is expected that the higher the early set-up cost, the less likely the users would adopt e-commerce. Similarly, Jati et al. (2017) studied the effect of cost and found that cost has a negative influence on e-commerce adoption. The micro agri-business enterprises in Longsheng, have little resources and capital, and too high-cost investment at the early stage of e-commerce adoption may impede the interest of the enterprises, thus, the cost may be considered a barrier to using e-commerce, and the following hypothesis is proposed.

***H1b**: Cost has a negative effect on the intention to adopt e-commerce in micro agri-business enterprises in Longsheng*.

#### Organization context

Organization readiness and technology readiness are selected to describe the organization context. Organization readiness reflects the organization's financial and human resource conditions for accepting the new technologies, as well as the organization's utilization level of creative information and skills (Vinod et al., 2018). Hamidreza (2012) and Walker et al. (2016) found that organization readiness has a positive effect on e-commerce adoption. Conversely, poor organization readiness manifests itself in financial constraints and a shortage of talent, which in turn will be a barrier to adoption. Therefore, the following hypothesis is proposed:

***H2a**: Organization readiness has a positive influence on the intention to adopt e-commerce in micro agri-business enterprises in Longsheng*.

Technology readiness is the basic technical infrastructure condition of the organization. To use the new technology, some infrastructure should be ready. If the organization has inadequate technology infrastructure, the intention to use the technology will be low. Rita and John (2015) and Carlo (2018) studied the significance of technology readiness and found that there is a positive effect on e-commerce adoption. Therefore, the following hypothesis can be given:

***H2b**: Technology readiness has a positive influence on the intention to use e-commerce in micro agri-business enterprises in Longsheng*.

#### Environment context

For the environment context, competitive pressure and government support are selected. Competitive pressure is used to reflect the pressure from the external environment, such as the competitors, suppliers, customers, industrial alliances, and partners. When a new technology appears, if the technology adopter gains profits and becomes a strong competitor, the potential adopters may have pressure and intention to use the new technology. In addition, if the suppliers, customers, industrial alliances, and partners prefer the new technology, the enterprise may have more serious pressure to digitalize its business. E-commerce has been used in more and more areas in China, and agri-business enterprises that fail to adopt e-commerce in Longsheng may face competitive pressure. Therefore, the following hypothesis is proposed:

***H3a**: Competitive pressure has a positive effect on the intention to use e-commerce in micro agri-business enterprises in Longsheng*.

Government support is the subsidy provided by the government to support the adoption of new technologies. Governmental support can attract users' attention to a certain extent and promote the adoption of new technologies. Seng et al. (2014) found that government support has a positive influence on e-commerce adoption. Raphael et al. (2016) found that government support has the greatest direct impact on intentions to use e-commerce. For agri-business enterprises in Longsheng, the government such as preferential policies on tax and financial services are established to support the development of e-commerce. Positive government support policy may increase the intention to use e-commerce to a certain extent. Therefore, the following hypothesis is proposed:

***H3b**: Government support has a positive effect on the intention to use e-commerce in micro agri-business enterprises in Longsheng, China*.

#### Entrepreneurial orientation

Abebe (2014) revealed in his study that the higher the EO, the higher the positive attitude toward new technology. In other words, the proclivity to behave entrepreneurially would facilitate the adoption of e-commerce. As explained earlier, EO in this study is understood as an owner's decision process that affects the enterprise's intention to innovate, be more proactive than its competitors, and take risks (Davis et al., 2010; Ade et al., 2020). Various studies have pointed out the inconsistent results of the different TOE factors on e-commerce adoption, such as the results from Jahongir and Shin (2014), Raphael et al. (2016), and Jati et al. (2017). This suggests that there may be some other factors moderating the relationship between the TOE factors and e-commerce adoption. Hence, the EO of the owners is modeled as a moderator in the relationship between the TOE factors and the intention to adopt e-commerce. The following hypothesis is proposed.

***H4**: The EO of owners positively moderates the relationship between perceived relative advantage, cost, organizational readiness, technology readiness, competitive pressure, and governmental support and the intention to adopt e-commerce in micro agri-business enterprises in Longsheng*.

Based on the seven hypotheses described above, a simplified representation of the conceptual framework model as presented in Figure 1 was tested.

## Research methodology and data analysis

In this part, the research methodology is given first, and then the data collection and PLS analysis process are explained in detail, and the regression equation is established to describe the relationship between TOE factors, EO, and intention to adopt e-commerce finally.

### Research methodology

The proposed research method involved a survey of owners among micro enterprises in Longsheng, China to investigate how the selected variables influence e-commerce adoption. Survey questionnaires were distributed to all the 337 micro agri-business enterprises registered in Longsheng's agricultural cooperatives record. The agricultural cooperatives are mutual economic organizations and they are set up to help Chinese traditional agri-business enterprises gain advantages in the system and break the scattered and unconsolidated weakness of family farming (Hoken and Su, 2018). As the agri-business enterprises are widely dispersed in the remote rural mountainous areas of Longsheng, a convenience sampling method, a non-probability sampling method was adopted since the target population is a homogenous population (Akram et al., 2020). To ensure representativeness, all the 337 micro enterprises have an equal opportunity to participate in the survey and online electronic questionnaires were mailed to all the owners of the micro enterprises.

### Data collection and analysis results

In this study, 214 questionnaires were collected, of which only 192 were valid questionnaires. The response rate is 56.9%. Among the 192 respondents, 92% have no college education and about 91% have an annual income of <200 thousand RMB, which is a relatively low income level. Therefore, most rural agri-business enterprises have low income and education levels, and they do not have sufficient resources and surplus funds for e-commerce development.

Furthermore, the structural equation modeling (SEM) analysis was undertaken to test the research hypotheses. Smart PLS was used for the PLS path modeling as the study seeks to examine the TOE theoretical framework and analyze the prediction power. In the following, the path model, the structural model with path analysis, and the assessment of the moderator with path analysis are explained successively.

#### Path model

**Figure 2:**
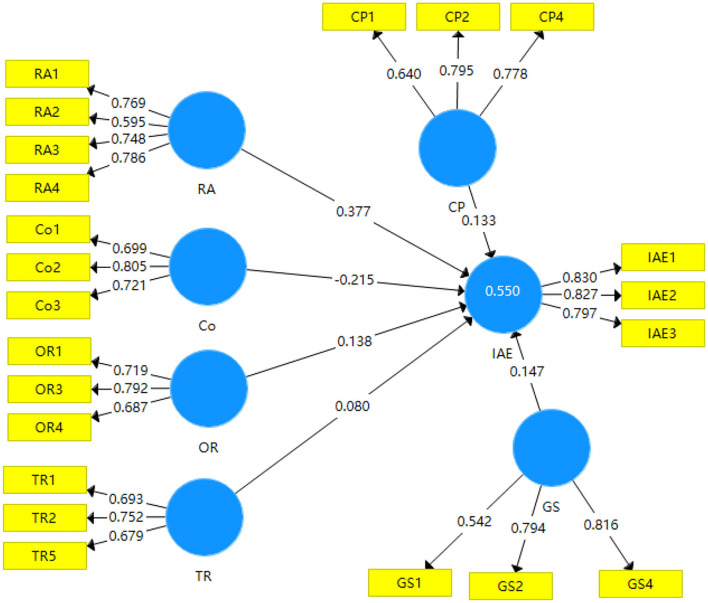
Path model after outer loading relevance testing.

The variables: relative advantage (RA), cost (Co), organization readiness (OR), technology readiness (TR), government support (GS), competitive pressure (CP), and intention to adopt e-commerce (IAE) were selected as the latent variables, and the related indicators were based on previously validated measurement scale as shown in Figure 2.

#### Structural model with path analysis and hypotheses testing

**Figure 3:**
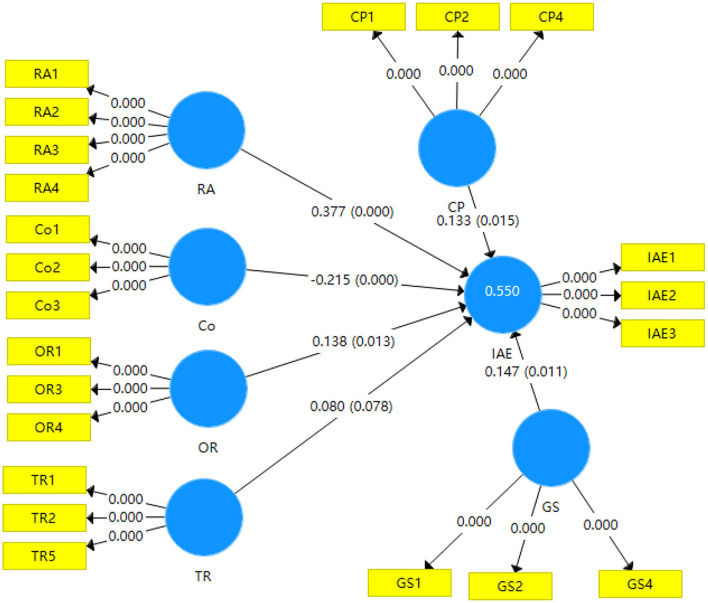
Path model after bootstrapping algorithm calculation with the p-value.

Path coefficients were calculated with bootstrapping algorithm to test the hypotheses and determine the causal relationship among the RA, Co, OR, TR, CP, GS, and IAE constructs. The significance level of 0.05 was assumed following the general convention (Hair et al., 2017). The results are shown in Figure 3.

The *p*-values of the relationships among RA, Co, OR, CP, GS, and IAE are lower than 0.05, and the causal relationships among them are all significant at a 5% significance level, so the hypotheses on the related TOE variables and IAE are validated. The causal relationship between TR and IAE is not significant at a 5% significance level, thus, the related hypothesis is not supported. Positive path coefficients among RA, OR, CP, GS, and IAE, and the related *p*-values are found to be lower than 0.05; hence the hypotheses H1a, H2a, H3a, and H3b are supported. In other words, RA, OR, CP, and GS all have a positive effect on IAE. The order of importance was determined by the path coefficient as follows: RA > GS > OR > CP. There is a negative path coefficient between Co and IAE, and the *p*-value is lower than 0.05, thus, hypothesis H1b is supported, that cost has a negative effect on IAE. There is a positive path coefficient between TR and IAE, but the *p*-value is higher than 0.05, thus, hypothesis H2b is not supported, and TR has no significant effect on IAE.

#### Assessment of moderator with path analysis and hypotheses testing

**Figure 4:**
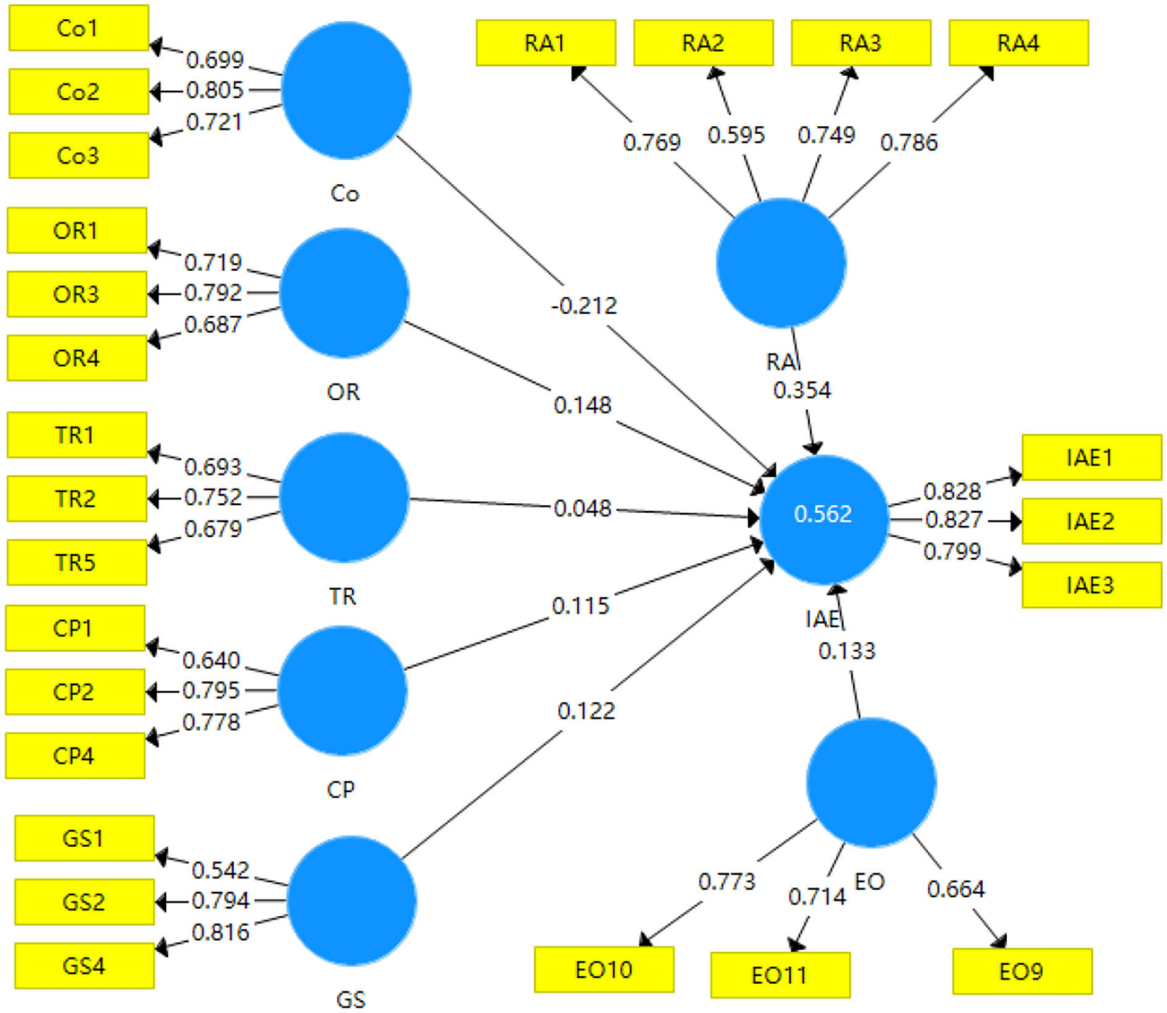
Path model with EO moderator outer loading relevance test.

To assess the moderating effect of EO on the relationship between TOE variables and IAE, a two-stage approach was used (Rigdon et al., 2010). In the first stage, the moderator was added to establish the measurement model and structural model as shown in Figure 4.

**Figure 5:**
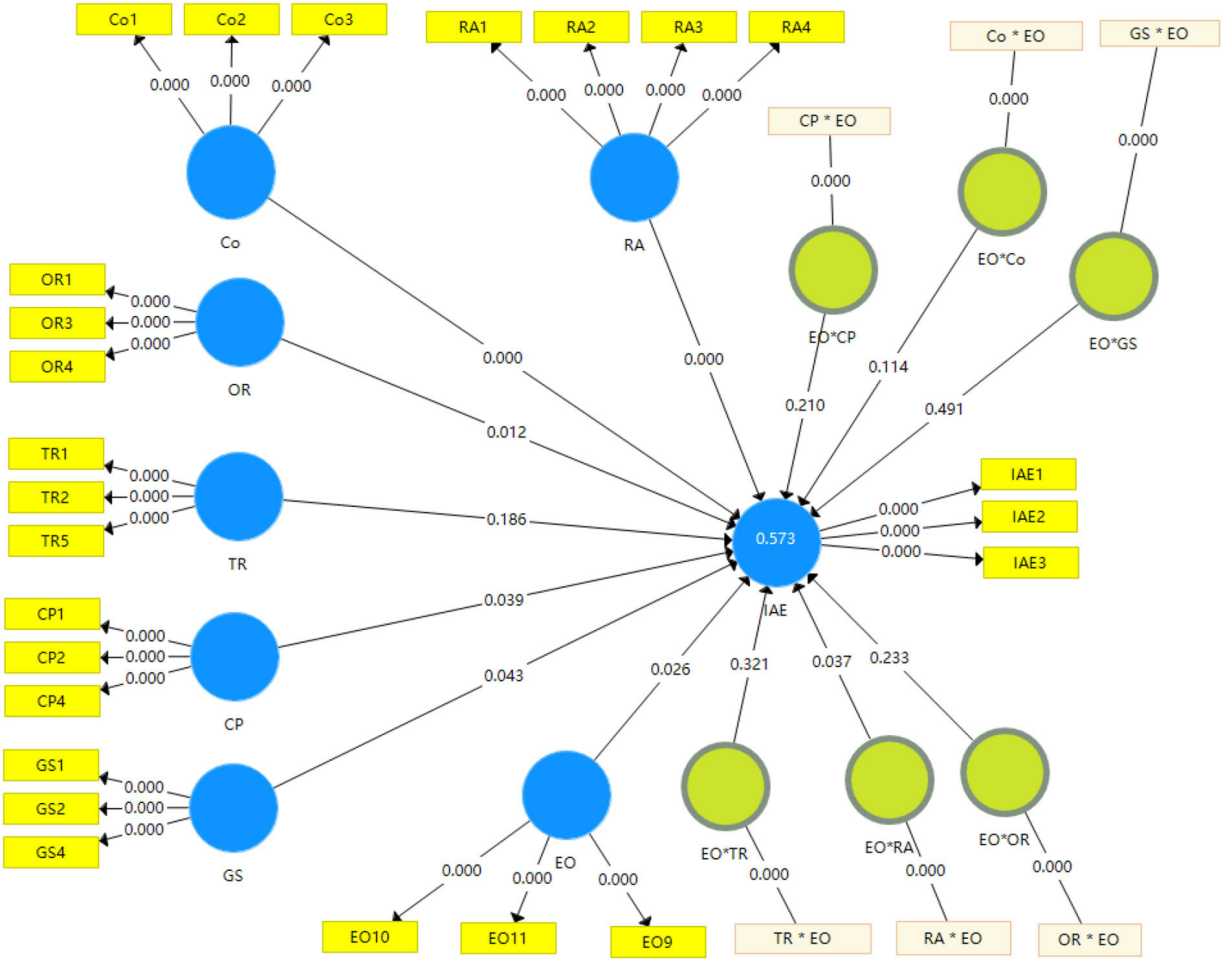
Measurement and structural model with EO and the interaction terms.

In the second stage, the TOE variables and moderator variables were multiplied to create a single-item measure to measure the interaction items as shown in Figure 5.

**Figure 6:**
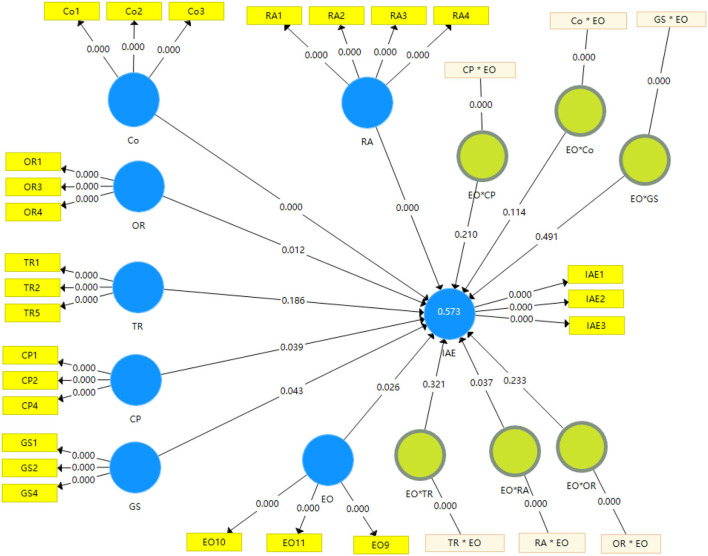
Moderating path model after bootstrapping algorithm calculation with the p-value.

With the calculation of the two stages, the significance of the moderating role on the relationships between the TOE variables and IAE was determined and the moderator hypotheses were tested. The calculation result of the moderating path model with six interaction items is shown in Figure 6. In Figure 6, the *p* values of the path coefficients of RA->IAE, Co->IAE, OR->IAE, CP->IAE, and GS->IAE are all below 0.05, thus, there are 5% level significance relationships between RA, Co, OR, CP, GS, and IAE. There are significant positive relationships among RA, OR, CP, GS, and IAE, and there is a significant negative relationship between Co and IAE, thus, hypotheses H1a, H1b, H2a, H3a, and H3b are all supported. The *p*-value of the path coefficients of TR->IAE is above 0.05, thus, there is no significant relationship between TR and IAE, and hypothesis H2b is not supported. The *p*-values of the path coefficients of EO^*^RA->IAE are below 0.05, thus, the moderator EO has a 5% level significance. Considered the path coefficient, EO has a significant positive moderating role on the relationship between RA and IAE, therefore, hypothesis H4a is supported. However, the *p*-values of the path coefficients of EO^*^Co->IAE, EO^*^OR->IAE, EO^*^TR->IAE, EO^*^CP->IAE, and EO^*^GS->IAE are all above 0.05, thus, the moderator EO has no significant moderating role on the relationships among Co, OR, TR, CP, GS, and IAE, and the hypotheses H4b, H4c, H4d, H4e, and H4f are all not supported.

In sum, the EO of owners has a significant positive moderating role only on the relationship between relative advantage and the intention to adopt e-commerce in agri-business enterprises in Longsheng, and EO has no significant moderating role on the relationships among cost, organization readiness, technology readiness, competitive pressure, government support, and the intention to adopt e-commerce in agri-business enterprises in Longsheng. The results are summarized as shown in Table 2.

**Table 2:** Main results on the TOE factors and EO moderator.

**Hypotheses**	**Path**	**Path coefficient**	***P*** **values**	**Results**
H1a: RA has a positive effect on IAE.	RA->IAE	0.373	0.000	H1a supported
H1b: Co has a negative effect on IAE.	Co->IAE	−0.223	0.000	H1b supported
H2a: OR has a positive effect on IAE.	OR->IAE	0.139	0.012	H2a supported
H2b: TR has a positive effect on IAE.	TR->IAE	0.056	0.186	H2b not supported
H3a: CP has a positive effect on IAE.	CP->IAE	0.113	0.039	H3a supported
H3b: GS has a positive effect on IAE.	GS->IAE	0.110	0.043	H3b supported
H4a: EO moderates positively the relationship between RA and IAE.	EO*RA->IAE	0.104	0.037	H4a supported
H4b: EO moderates positively the relationship between Co and IAE.	EO*Co->IAE	0.057	0.114	H4b not supported
H4c: EO moderates positively the relationship between OR and IAE.	EO*OR->IAE	−0.041	0.233	H4c not supported
H4d: EO moderates positively the relationship between TR and IAE.	EO*TR->IAE	0.027	0.321	H4d not supported
H4e: EO moderates positively the relationship between CP and IAE.	EO*CP->IAE	−0.050	0.210	H4e not supported
H4f: EO moderates positively the relationship between GS and IAE.	EO*GS->IAE	0.002	0.491	H4f not supported

**Figure 7:**
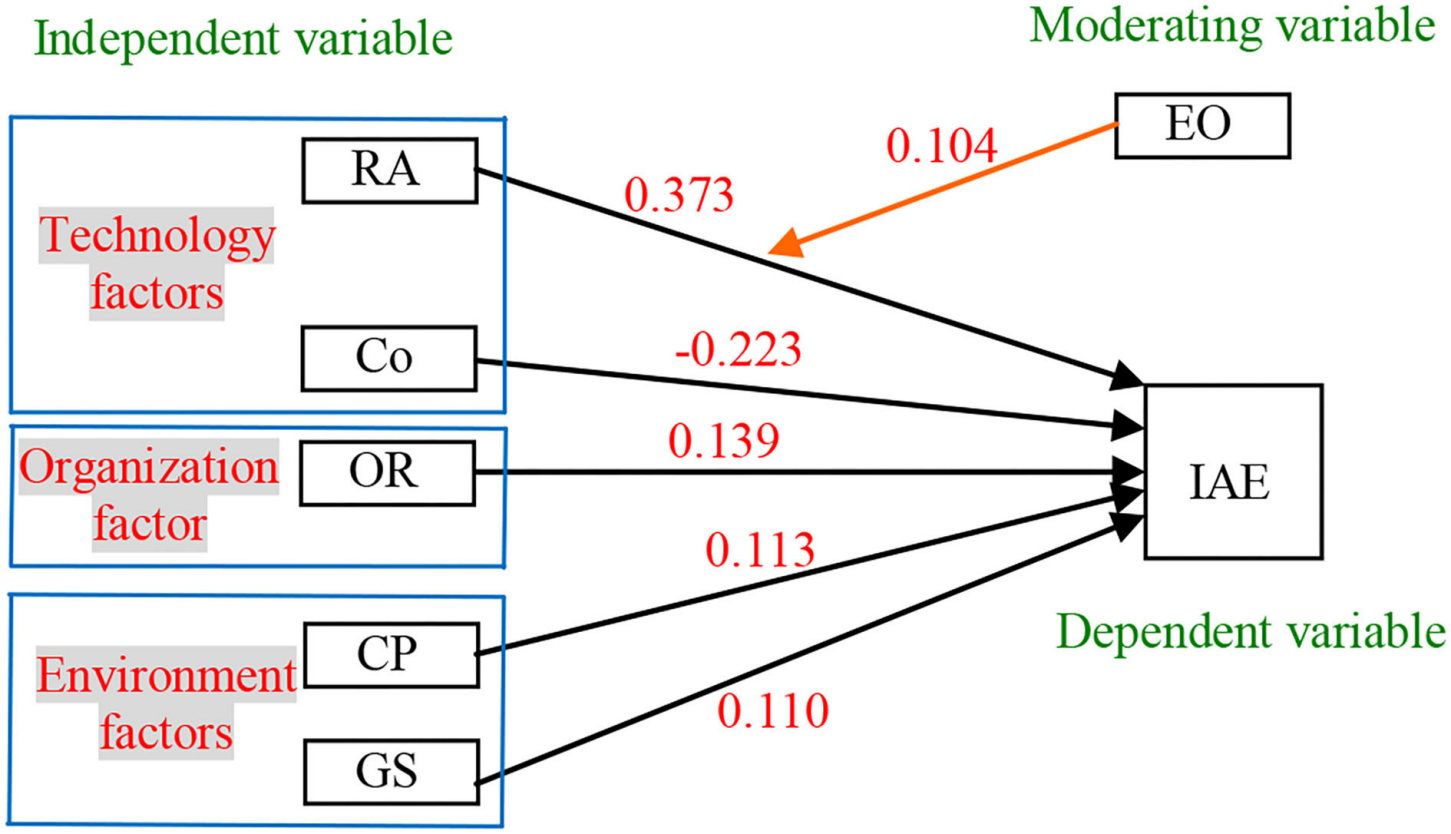
The extended TOE framework after the hypotheses test.

With the hypotheses test, the relationships are described as per the extended TOE framework shown in Figure 7.

In the extended TOE framework, RA has a positive influence on IAE with a path coefficient of 0.373, and Co has a negative influence on IAE with a path coefficient of−0.223. OR is the organization factor, and it has a positive influence on IAE with a path coefficient of 0.139. The environment factors include CP and GS, and they both have a positive influence on IAE with path coefficients of 0.113 and 0.110. EO, as a moderating variable, has a positive moderating role in the relationship between RA and IAE with a path coefficient of 0.104. Based on the path coefficient in Figure 7, the regression equation can be established as:


(1) $$\begin{array}{l} \begin{array}{c} IAE=\left(0.373+0.104\;⁎\;EO\right)\;⁎\;RA-0.223\;⁎\;Co\\ \;+0.139\;⁎\;OR+0.113\;⁎\;CP+0.110\;⁎\;GS \end{array} \end{array}$$

In the equation, IAE is related to RA, Co, OR CP, GS, and EO^*^RA linearly and the relationship between IAE and RA is moderated with EO. RA, OR, CP, and GS have a positive effect on IAE, and Co has a negative effect on IAE. EO moderates positively the relationship between RA and IAE.

## Discussion of the key findings

Relative advantage is regarded as the most important factor influencing the adoption intention of e-commerce in these micro enterprises. This finding is not unexpected as the e-commerce platform allows enterprises to use pictures, live streaming, and run marketing campaigns to drive sales. In addition, with e-commerce, enterprises can reach new markets and information and charge higher prices for their produce. This can motivate a shift from the traditional channel of selling through intermediaries which are perceived to bring the cost down and give a higher margin for their produce. Furthermore, e-commerce is perceived to provide them with access to a larger market, and not having to rely on intermediaries such as wholesalers which may allow them to sell directly at a higher price. The results validate prior studies such as Liu and Ju (2013) and Rita and John (2015) that relative advantage was a significant predictor for adopting e-commerce in SMEs. Conversely, the initial cost of implementing e-commerce was found to negatively affect the intention to adopt e-commerce. This result is not surprising as many micro enterprises have very limited financial resources. The cost of adopting, maintaining, and supporting e-commerce as well as training employees to use e-commerce technologies is high relative to their annual income. Small and micro enterprises have annual taxable income of up to 3 million RMB (US$ 460,000) and many are crumbling as material cost soars and surviving on Chinese government support in the forms of tax cuts, extension of loan support scheme, and credit support. The negative influence of cost on technology adoption is well supported by many studies (Adi and Sudarsono, 2016; Jati et al., 2017).

Technology readiness was found to have no significant effect on the intention to adopt e-commerce. This result is somehow unexpected. Many are already familiar with the Taobao e-commerce platform as users, thus, there is no issue accepting the innovation of e-commerce technology. Taobao e-commerce platform is also designed to appeal to local users' preferences, such as the instant messaging tool of Aliwangwang to facilitate buyer-seller communication, and a payment tool of Alipa, which offers both security and speed, is very popular with consumers from all walks of life. Therefore, most rural enterprises may not consider technology readiness as an impediment to e-commerce adoption in Longsheng. The organization readiness has a positive impact on the intention to adopt e-commerce. The finding is consistent with past research on the significant influence of organization readiness on the adoption of new technology such as studies by Hamidreza (2012) and Walker et al. (2016). In Longsheng, most rural agri-business enterprises have income below 200,000 RMB per year and low education levels, and they do not have sufficient resources and surplus funds for e-commerce development. Hence, organizational readiness would be a major concern for these rural agri-business enterprises.

The competitive pressure has a positive influence on the intention to adopt e-commerce for agri-business enterprises in Longsheng. This finding supports previous findings (Rita and John, 2015; Ahmed, 2018; Rohit and Tripti, 2019). In Longsheng, the mountainous area accounts for 87.2% of the county, and most agri-business enterprises are in the remote mountain areas, e-commerce enhances their competitive advantage by increasing the speed of doing business and this can provide a powerful competitive advantage to those using e-commerce as a platform to merchandise their products. Thus, competitive pressure is undoubtedly one of the key factors that drive the adoption of e-commerce in rural China. Similarly, government support has a positive influence on the intention to adopt e-commerce and the finding is consistent with past works of literature (Seng et al., 2014; Raphael et al., 2016). Government intervention such as building infrastructure and reducing network costs is especially important in sustaining technological development in small and micro enterprises. Hence, government support to provide the infrastructure, finance, and information for the development of e-commerce, is key for a backward rural county such as Longsheng.

On the interactive effect of EO, only relative advantage was found to have a significant effect. This implies that most micro business owners do not demonstrate EO which results in low EO at the organizational level. These counterintuitive results can best be explained by the demographic characteristics of the respondents. This low EO among the owners of agri-business enterprises in Longsheng has contributed to the weakening of the relationship between the TOE factors and the intention to adopt e-commerce. These findings suggest that to improve the uptake of e-commerce among the rural micro agri-business enterprises, it is important to cultivate the business owners' EO consistent with the RBW theory that EO is made up of resources and capabilities and can serve as a mechanism, the resource that represents the owners' processes, habits and decision-making styles that help them to function entrepreneurially in managing their e-commerce. Owners with strong entrepreneurial orientation have more resources allocated for innovation to gain a competitive advantage related to daily activities (Bai and Ren, 2016; Ha et al., 2018).

## Conclusion, limitation, and future research

This study advances research on e-commerce by highlighting the importance of the individual agri-business owners' EO as a moderating variable in the relationship between the TOE factors and e-commerce adoption. EO as a moderator is found to have higher predictive power than that of TOE factors. The effect size of all the TOE factors except for technology readiness is moderate. It implies these factors may not necessarily pose a threat to e-commerce adoption among non-adopters. Hence, it is reasonable to conclude that regardless of the benefits and advantages that e-commerce may bring to enterprises, without EO, agri-business enterprises will be less willing to adopt e-commerce as they have low risk propensity, less proactiveness, and innovativeness. This study suggests that there are opportunities for developmental efforts to promote an entrepreneurial mindset among micro businesses owners in rural China as the low EO among farmers may impede the adoption of e-commerce in rural China. Micro agri-business enterprises would benefit from participating in entrepreneurial practice activities organized by the government and the e-commerce platform providers. Policy makers should also design special programs to support farmers to be more entrepreneurial, especially in adapting the market changes and facilitating these micro business farmers to have broader access to export markets or modern food retails, by improving farmers' capacity in capturing opportunities. In addition, the rural e-commerce training system can be established to provide skills training and entrepreneurial practice activities for returning rural migrant workers, college students, veterans, poor households, and other potential business owners of micro agri-business enterprises. This would help to cultivate the EO qualities of these owners to help them learn new modes of business and management, broaden their minds, and develop the confidence to try new technologies.

There are several limitations of this study. First, the selection of the study's respondents using the convenience sampling method is confined to only micro businesses registered with Longsheng's agricultural cooperatives. This may cause over or under representation of the population as some of the agri-business enterprises are not registered with the cooperatives. Therefore, the generalization of results to other alternate settings or businesses not registered with the cooperatives may be not applicable. Second, there are only 337 agri-business enterprises in Longsheng which are relatively small. In the future, multiple counties of cities should be considered. Finally, the study is confined to testing the relationships among TOE factors and intention to adopt e-commerce in rural China; there are many other factors that were not considered, such as social media, logistic networks, security issues, age, sex, education level, and other economic and external factors. Sohaib et al. (2022) found that sex has an influence on the decision-making of the owners. Wang and Muhammad (2022) found that education has a positive influence on subjective wellbeing. Future research can replicate this study in a wider scope by adding more variables to better comprehend the development of e-commerce in rural China. Furthermore, among all the variables tested in the research model of the current study, the analysis showed the moderating effect of EO on relative advantage and not the other TOE factors. Hence, factors contributing to the moderating effect of EO on e-commerce adoption should be investigated in a more holistic approach by integrating the demographic variables of the owners. Findings from this study can be replicated in other developing countries in Southeast Asia such as Laos, Indonesia, and Myanmar having similar conditions to see if there is a significant difference between them. Additionally, the case study research method can be used on selected enterprises that have adopted e-commerce to provide deeper insights into the best practices, challenges, and barriers slowing down their implementation of e-commerce. In addition, national and organization cultures can be added to study their influence on e-commerce adoption.

## Data availability statement

The raw data supporting the conclusions of this article will be made available by the authors, without undue reservation.

## Author contributions

Material preparation, data collection, and analysis were performed by LH and LG. The first draft of the manuscript was written by LH, LG, and YS. All authors commented on previous versions of the manuscript, contributed to the study's conception and design, read, and approved the final manuscript.

## Conflict of interest

The authors declare that the research was conducted in the absence of any commercial or financial relationships that could be construed as a potential conflict of interest. The reviewer K-YL declared a shared affiliation with the authors LH and YS to the handling editor at the time of review.

## Publisher's note

All claims expressed in this article are solely those of the authors and do not necessarily represent those of their affiliated organizations, or those of the publisher, the editors and the reviewers. Any product that may be evaluated in this article, or claim that may be made by its manufacturer, is not guaranteed or endorsed by the publisher.
